# Determinants and socioeconomic inequalities in vitamin A-rich food consumption among children aged 6–23 months in Somalia: a multilevel analysis of the 2020 Somalia Health and Demographic Survey (SHDS)

**DOI:** 10.3389/fnut.2026.1809278

**Published:** 2026-05-18

**Authors:** Asma Mahamoud Abdillahi, Abdirizak Hassan Abokor, Omer Adam Farih, Mustafe Abdillahi Ali, Saeed Hassan Mohamed, Ahmed Adan Abdi, Awale Ali Omer, Hafsa Mohamed, Abdisalam Hassan Muse, Hodo Abdikarim

**Affiliations:** 1 Research and Innovation Center, Amoud University, Borama, Somalia; 2 Institute of Social Science, Selçuk University, Konya, Türkiye; 3 College of Medicine and Health Sciences, University of Hargeisa, Hargeisa, Somalia; 4 Gabiley Mental Hospital, Gabiley, Somalia

**Keywords:** Vitamin A, multilevel analysis, Somalia, dietary intake, concentration index

## Abstract

**Introduction:**

Vitamin A deficiency (VAD) remains a public health concern, particularly among children in resource-limited settings. This study aimed to evaluate the consumption of vitamin A-rich foods and associated socioeconomic inequalities among children aged 6–23 months in Somalia.

**Methods:**

Data from the 2020 Somalia Health and Demographic Survey (SHDS were analyzed, including a sample of 1,839 children. Multilevel binary logistic regression was used to identify determinants of vitamin A-rich food consumption. Concentration indices (CIX) were employed to assess socioeconomic inequalities. Statistical analyses were conducted using Stata 17 and R Studio.

**Results:**

Only 29.7% of children consumed vitamin A-rich foods. Significant regional variations were observed. Multilevel analysis revealed that household wealth (*AOR* = 3.60, 95% CI: 2.23–5.78 for highest vs. lowest quintile), media access (*AOR* = 2.05, 95% CI: 1.41–2.98), paternal education (*AOR* = 1.83, 95% CI: 1.35–2.49), and antenatal care (ANC) attendance (*AOR* = 1.69, 95% CI: 1.09–2.62 for ≥1 visit) were significantly associated with vitamin A-rich food consumption. The relative CIX indicated substantial wealth-related (0.2533, *p* < 0.001) and education-related (0.1438, *p* < 0.001) inequalities.

**Conclusion:**

Vitamin A-rich food consumption among young children in Somalia is low, with significant socioeconomic inequalities. Interventions should target low-income households, promote maternal education, improve access to media, and strengthen antenatal care services to enhance vitamin A intake. Furthermore, regional-specific interventions to promote vitamin A food are recommended.

**Recommendations:**

Policymakers should prioritize nutrition-sensitive social protection programs, strengthen antenatal care services, and implement targeted behavior change communication to improve vitamin A-rich food consumption. Vitamin A supplementation programs must be strengthened to ensure high coverage among children aged 6–59 months, especially in regions with the lowest intake. Future research should focus on longitudinal studies to assess the long-term impact of these interventions.

## Introduction

Vitamin A is an essential micronutrient required in trace amounts for healthy growth and development, immune system function, reproduction, and the cellular integrity of epithelia (1, 2). Vitamin A can be found in diet in two forms: provitamin A carotenoids and preformed vitamin A (3). The majority of preformed vitamin A is found in animal products, including whole milk, egg yolks, glandular meats, liver, and fish liver oils, as well as human milk. Green leafy vegetables (including spinach, amaranth, and young leaves from other sources), yellow vegetables (like carrots, avocados, and squash), and orange and yellow non-citrus fruits (such papayas, mangoes, and apricots) are all good sources of provitamin A carotenoid. Micronutrient status can have a direct or indirect impact on health outcomes like child survival, growth, and development through interactions that increase food intake due to an enhanced appetite and lower morbidity (4).

Research carried out in underdeveloped nations like Bangladesh and India revealed that preschool-aged children (1–3 years old) had low dietary vitamin A intakes and that the majority of their non-breast milk vitamin A intake came from plant sources (5, 6). Since newborns have low vitamin A reserves at birth, and these reserves are significantly lower when the mother is vitamin A deficient, supplemental foods should supply sufficient levels of vitamin A, particularly after the age of 4–6 months (7). Eating at least one vitamin A-rich food item from the primary food category is considered adequate vitamin A consumption (8).However, ensuring vitamin A is accessible in sufficient quantities continues to be difficult, particularly in nations with low resources. Over 124 million children are thought to need access to vitamin A-rich diets or supplements worldwide (9).

Unlike broader dietary diversity indicators, vitamin A deficiency is a specific, high-burden public health problem in Somalia, directly contributing to mortality from measles, diarrhea, and malaria among children under five (9, 10). Because vitamin A cannot be synthesized by the human body and must be obtained through diet (7, 8), identifying barriers to consuming vitamin A-rich foods is essential for designing effective supplementation and dietary diversification programs. While minimum dietary diversity is an important metric, focusing specifically on vitamin A allows for direct alignment with existing national supplementation programs and provides actionable insights for a deficiency that remains a leading cause of preventable childhood blindness and mortality in low-resource settings.

Vitamin A-rich foods and other micronutrients are not consumed in the majority of African nations at the daily recommended level (11–13). In the majority of low- and middle-income nations children in the households in the lowest wealth quintile as defined by the SHDS wealth index constructed using principal component analysis of household assets and housing characteristics have a disproportionately high burden of vitamin A deficiency (VAD) and consume less foods high in VA (14, 15).

VAD, a prevalent micronutrient deficiency (16), has been exacerbated by poverty and a higher incidence of infectious diseases (17). In African nations, it is the primary cause of measles, diarrhea, and malaria-related morbidity and mortality in children aged 6 to 59 months (9–11, 18). In addition, VAD produces Bitot's spots, night blindness, and other pathological disorders (19). Inadequate intake of foods high in VA contributes to the high incidence of VAD (20).

There are socioeconomic disparities in children's access to micronutrients, and some studies have identified the factors that contribute to these disparities in low-resource nations (21–24). To determine the potential contributing causes to the observed inequality of a given health outcome and to assess the degree of inequality of that outcome, the concentration index and the Wagstaff decomposition analysis are the suitable statistical methods (25).

Accordingly, some of the major factors influencing the socioeconomic disparities in access to vitamin A consumption in particular and micronutrients in general are women's educational attainment, income, age, marital status, occupation, place of residence, use of prenatal and antenatal care, child age, sex, current breastfeeding status, birth order, and number of children under five in the family (15, 23, 24, 26, 27). Therefore, one of the most important steps in enhancing children's general health and wellbeing is identifying and minimizing preventable causes of socioeconomic disparities in access to vitamin A-rich foods (22).

Supplementation programs, dietary changes, and other local settings are effective ways to prevent micronutrient deficiencies, including the VAD (10, 28, 29). Interestingly, vitamin A supplementation is a successful tactic that has demonstrated a noteworthy decrease in the morbidity and mortality associated with vitamin A deficiency illness across several nations (30, 31). However, it only helps to achieve minimally adequate vitamin A storage during the first 2 years of life, therefore it does not sustainably improve a population's vitamin A status (29). Therefore, the most effective way to reduce the burden of VAD is to think about dietary changes that result in the consumption of foods high in vitamin A (32).

Low levels of vitamin A, zinc, and iron in Somali children weaken their immune systems and increase their risk of infection. The risk of death from malaria, measles, and diarrhea is greatly increased by insufficient vitamin A intake. A zinc shortage is linked to a higher risk of death from malaria, pneumonia, and diarrhea (33). Additionally, compared to 22% of toddlers aged 6–23 months who reported eating foods high in iron, only 33% reported eating meals high in vitamin A. Eighteen percent of children of this age had fruits and vegetables rich in vitamin A. Children who lived in urban areas were the most likely to receive vitamin A supplements (48 percent), followed by those who lived in rural areas (39 percent), while children who were nomadic received the lowest percentage (14%) (34).

Despite the high burden of VAD in Somalia, no previous study has comprehensively examined the determinants and socioeconomic inequalities in vitamin A-rich food consumption using advanced methods. Therefore, this study aimed to (1) estimate the magnitude of vitamin A-rich food consumption among children aged 6–23 months, (2) identify individual- and community-level factors associated with consumption using multilevel binary logistic regression, and (3) quantify wealth- and education-related inequalities using the concentration index. The findings will inform targeted, equity-focused nutrition interventions and guide policymakers in prioritizing resources to regions and populations with the greatest need. Wealth and education were selected for inequality analysis because wealth is a key determinant of food affordability and access to diverse nutrient-rich diets, with prior studies in LMICs consistently showing wealth-based disparities in child nutrition; similarly, maternal education influences nutrition knowledge, health-seeking behavior, and child feeding practices, and is a modifiable factor for policy interventions.

## Method

### Study design and data source

The data for this study were obtained from the 2020 Somalia Health and Demographic Survey (SHDS), a nationally representative survey conducted by the Ministry of Planning, Investment and Economic Development in cooperation with the Ministry of Health and Human Services of the Federal Government of Somalia. The SHDS was conducted across 16 pre-war geographical regions of Somalia, except for two regions excluded due to security concerns. These surveys provided comprehensive data on population characteristics, maternal and child health, fertility, mortality, nutrition, and other critical indicators (35–41). A stratified, multi-stage cluster sampling design was used to ensure national and regional representativeness. The sampling frame included urban, rural, and nomadic populations, with Enumeration Areas (EAs) selected using high-resolution satellite imagery. The SHDS employed a three-stage sampling approach, selecting Primary Sampling Units (PSUs) and Secondary Sampling Units (SSUs) proportionally to size, followed by systematic household sampling. In total, the survey included 15,870 households, with 16,715 women interviewed. The SHDS sampled ever-married women aged 15–49 years. Our analytic sample was restricted to currently married women with children aged 6–23 months because the detailed child feeding module (including the 24-h dietary recall for vitamin A-rich foods) was administered only to currently married mothers who resided with their child. This restriction was consistent with standard DHS methodology and ensured accurate matching of maternal and child data. For this study, the dataset was cleaned and preprocessed using Stata 17, with missing observations removed. The final sample comprised 1,839 children aged 6–23 months born to married women within the 5 years preceding the survey, ensuring a focused analysis of vitamin A-rich food intake among young children in Somalia.

## Study variables

### Outcome variable

The outcome variable for this study was Vitamin A-rich food consumption among children aged 6–23 months. This binary variable was coded as “1” if the child had consumed at least one Vitamin A-rich food item in the 24 h preceding the survey and “0” otherwise. The food items considered as Vitamin A-rich included eggs, meat (beef, lamb, chicken), fish or shellfish, liver and other organ meats, dark green leafy vegetables, pumpkin, carrots, squash (yellow or orange inside), and Vitamin A-rich fruits such as mangoes and papayas.

### Explanatory variables

The selection of explanatory variables was based on subject knowledge and previous studies (35) and included residential factors comprised region (16 regions in Somalia) and residence type (urban, rural, and nomadic). Maternal factors included mother's age (15–19, 20–24, 25–29, 30–34, 35–39, 40–49 years), mother's education level (no education, primary, secondary/higher), number of ANC visits during pregnancy (zero, one, two, three or more), place of delivery (home, health center—both private and public), currently breastfeeding status (yes, no), and media exposure (yes if the mother listens to the radio, watches television, or both; no if neither). Household and husband-related factors included the household head's sex (male, female), household head's age (less than 21, 21–30, 31–40, 41–50, 51 or above), wealth index (lowest, second, middle, fourth, highest), total children ever born (1–3, 4–6, 7 or more), and husband's education status (ever attended school: yes, no). Child-related factors included birth order (firstborn, second to fourth, fifth or more), sex of the child (male, female), and child's age in months (6–23).

Although ‘total children ever born' (parity) and ‘birth order' are correlated, they capture distinct constructs. Parity reflects cumulative maternal reproductive experience and household resource dilution (larger families may have fewer resources per child). Birth order captures the child's position relative to siblings, allowing us to test whether first-born children receive different feeding practices compared to later-born children due to parental inexperience or shifting priorities. Both variables were retained to avoid omitted variable bias, as prior studies have shown they can have independent effects on child nutrition (15, 26).

The wealth index is a composite measure of household socioeconomic status constructed by the SHDS using principal component analysis (PCA) of household assets (e.g., ownership of television, bicycle, car), housing characteristics (e.g., flooring material, water source, toilet facility), and access to services. Households were assigned scores and then divided into quintiles (lowest, second, middle, fourth, highest) based on the national distribution

## Selection of covariates and hypotheses

Covariates were selected based on the UNICEF conceptual framework of child nutrition and prior studies from low- and middle-income countries (15, 23, 24, 26, 27). Table 1 summarizes each variable, its rationale, and the hypothesized association. For example, we hypothesized that higher household wealth would be positively associated with vitamin A-rich food consumption due to increased affordability of animal-source foods and fruits; that maternal education would have a positive effect through improved nutrition knowledge and health-seeking behavior; and that ANC attendance would be positively associated due to nutrition counseling opportunities during visits. Community-level variables (e.g., low community literacy) were hypothesized to exert contextual effects beyond individual-level factors, reflecting social norms and access to health infrastructure.

**Table 1:** Selection of covariates and hypotheses.

Variable category	Variable name	Rationale for inclusion (based on UNICEF framework & prior LMIC studies)	Hypothesized association with vitamin A-rich food consumption
Child-level factors	Child's age (months)	Older children are introduced to a wider variety of complementary foods; feeding practices evolve with age (15, 35).	Positive (+): older children (12-23 months) more likely to consume VA-rich foods than infants (6–11 months).
	Sex of child	Gender-based feeding discrimination has been documented in some settings, though evidence is mixed (26)	Neutral or uncertain: no strong directional hypothesis; we will explore this empirically.
	Birth order	First-born children may receive more attention, while later-born children may benefit from parental experience (15, 26).	Positive (+): higher birth order associated with greater consumption due to accumulated maternal experience.
	Currently breastfeeding	Breastfeeding may delay introduction of complementary foods; non-breastfed children require diverse solid foods earlier (35).	Positive (+): non-breastfed children more likely to consume VA-rich foods.
Maternal factors	Mother's age	Younger mothers may have less nutrition knowledge and feeding experience; older mothers may have established practices (23).	Positive (+): older maternal age associated with higher consumption.
	Mother's education	Education improves health literacy, nutrition knowledge, information-seeking behavior, and healthcare utilization (15, 24, 27).	Positive (+): higher maternal education associated with greater VA-rich food consumption.
	Media access (radio/TV)	Media exposure delivers nutrition messages, health campaigns, and behavior change communication (26).	Positive (+): media access associated with higher consumption.
	Number of ANC visits	ANC provides platform for nutrition counseling, supplementation, and health messaging during pregnancy (26, 27).	Positive (+): any ANC attendance associated with higher consumption; dose-response expected.
	Place of delivery	Facility delivery may increase contact with health workers who provide feeding advice; may proxy for healthcare access (35).	Positive (+): health facility delivery associated with higher consumption.
Household/husband factors	Wealth index (quintiles)	Higher wealth enables purchase of diverse, nutrient-dense foods (animal products, fruits, vegetables); affordability is a key barrier (15, 23, 36).	Positive (+): higher wealth quintile associated with substantially higher consumption.
	Husband's education	Educated husbands may support better household nutrition, share childcare responsibilities, and have higher income (23).	Positive (+): paternal education associated with higher consumption.
	Household head's sex	Female-headed households may have different resource allocation priorities; evidence is mixed across settings (15).	Neutral or uncertain: explored empirically.
	Household head's age	Older household heads may have more stable income and experience, or may be more traditional (23).	Positive (+): older age associated with higher consumption.
	Total children ever born (parity)	Higher parity may lead to resource dilution (fewer resources per child) or accumulated maternal experience (15, 26).	Uncertain: competing directions; explored empirically.
Community-level factors	Community media exposure	Social norms around media use; clusters with high media access may have better diffusion of nutrition messages (35).	Positive (+): high community media access associated with higher consumption (contextual effect).
	Community education (literacy)	Collective health literacy; clusters with educated mothers create supportive norms for child feeding (35, 36).	Positive (+): high community literacy associated with higher consumption (contextual effect).
	Community poverty	Concentrated poverty limits food availability, market access, and nutrition program reach (36).	Negative (–): high community poverty associated with lower consumption (contextual effect).
Geographic factors	Region (16 regions)	Regional variations in food systems, agricultural production, market access, conflict, health infrastructure, and cultural practices (34).	Significant variation expected; some regions (e.g., Awdal, Bakool) hypothesized to have higher consumption than others (e.g., Togdheer, Sool).
	Type of residence (urban/rural/nomadic)	Urban areas typically have better market access and health services; nomadic populations face unique mobility constraints (34).	Positive (+): urban residence associated with highest consumption; nomadic with lowest.

## Statistical analysis

Statistical analyses were conducted using Stata 17 and R Studio. The analysis began with a proportion table for the dependent variable (DV), followed by a univariate analysis to examine the frequencies and percentages of predictor variables. Bivariate analysis was then performed using Chi-square tests to assess associations between the DV and independent variables. The Concentration Index (CIX)—both Relative CIX and Modified CIX—along with the Concentration Curve, were utilized to assess disparities in vitamin A-rich food consumption among children aged 6–23 months, based on wealth index and maternal education (36–38). Due to the hierarchical nature of the DHS data, where individuals (Level 1) are nested within Primary Sampling Units (PSUs/Clusters, Level 2), a standard logistic regression would violate the assumption of independence. Such nested structures often exhibit intra-cluster correlation, where observations within the same cluster are more similar than those across different clusters. To account for this, a multilevel binary logistic regression was conducted. The model, which estimates the relationship between predictors and the probability of the outcome while adjusting for cluster-level variance, is expressed as follows (1):


$$\text{logit}\left({\text{P}}_{\text{ij}}\right)={\text{γ}}_{00}+\sum_{\text{p}}{\text{γ}}_{\text{p}}{\text{X}}_{\text{pij}}+\sum_{\text{q}}{\text{γ}}_{\text{q}}{\text{Z}}_{\text{qj}}+{\text{u}}_{0\text{j}}$$ (1)

In this model, **P**_**ij**_ represents the probability of the outcome for individual *i* in cluster *j*, while *logit*(*P*_*ij*_) denotes the corresponding log-odds of the outcome. The term γ_00_ refers to the overall intercept, which reflects the average log-odds of the outcome when all predictors are held at zero. The variables *X*_*pij*_ represent individual-level characteristics, such as age and education, whereas *Z*_*qj*_ denote community-level factors, including region and place of residence. The coefficients γ_*p*_ capture the effects of individual-level variables, and γ_*q*_ represent the effects of community-level variables. Finally, *u*_0*j*_ is the random effect at the cluster level, accounting for unobserved variation between clusters. The analysis incorporated four models: Model 0 (baseline model), Model I (individual-level predictors), Model II (community-level factors), and Model III (combined individual and community-level factors). Model fit was assessed using the Akaike Information Criterion (AIC) and Bayesian Information Criterion (BIC), where lower values indicated a better fit. Log-likelihood and variance were used to evaluate model performance, while Intraclass Correlation Coefficient (ICC) measured variation at the group level. Finally, the study accounted for the DHS complex survey design by applying sampling weights using the svy command for primary analyses. In the multilevel models, weights were incorporated to address the hierarchical structure of the data and the statistical significance was set at *p* < 0.05 throughout the analysis.

Community-level variables were created by aggregating individual characteristics at the cluster level (V001). For each variable, proportions were calculated within clusters using binary indicators, and then dichotomized based on the mean value to classify clusters. Specifically, community media exposure was derived from the proportion of individuals with no media exposure, with clusters below the mean categorized as high media exposure and those above as low media exposure. Community poverty was constructed from the proportion of households in the poorest and poorer wealth quintiles, with clusters above the mean classified as high poverty and those below as low poverty. Community education was based on the proportion of women with no formal education, where clusters above the mean were labeled as low literacy and those below as high literacy.

## Magnitude of vitamin A rich food consumption among children aged 6–23 months

This study analyzed data from 1,839 children using the 2020 Somalia BDHS dataset. The findings, based on weighted descriptive analysis as shown in Table 2, indicated that 30.2% (95% CI: 27.6%−33%) of children aged 6–23 months consumed at least one vitamin A-rich food in the 24 h preceding the survey, while 69.8% (95% CI: 67%−72.4%) did not. The relatively small standard error (0.014) suggested that the sample proportion was a precise estimate of the population proportion.

**Table 2:** Magnitude of vitamin A rich food consumption among children aged 6–23 months.

Outcome variable	Proportion	Standard error	95% confidence interval	
*n* = 1,839				
No	0.698	0.014	0.670	0.724
Yes	0.302	0.014	0.276	0.330

**Figure 1:**
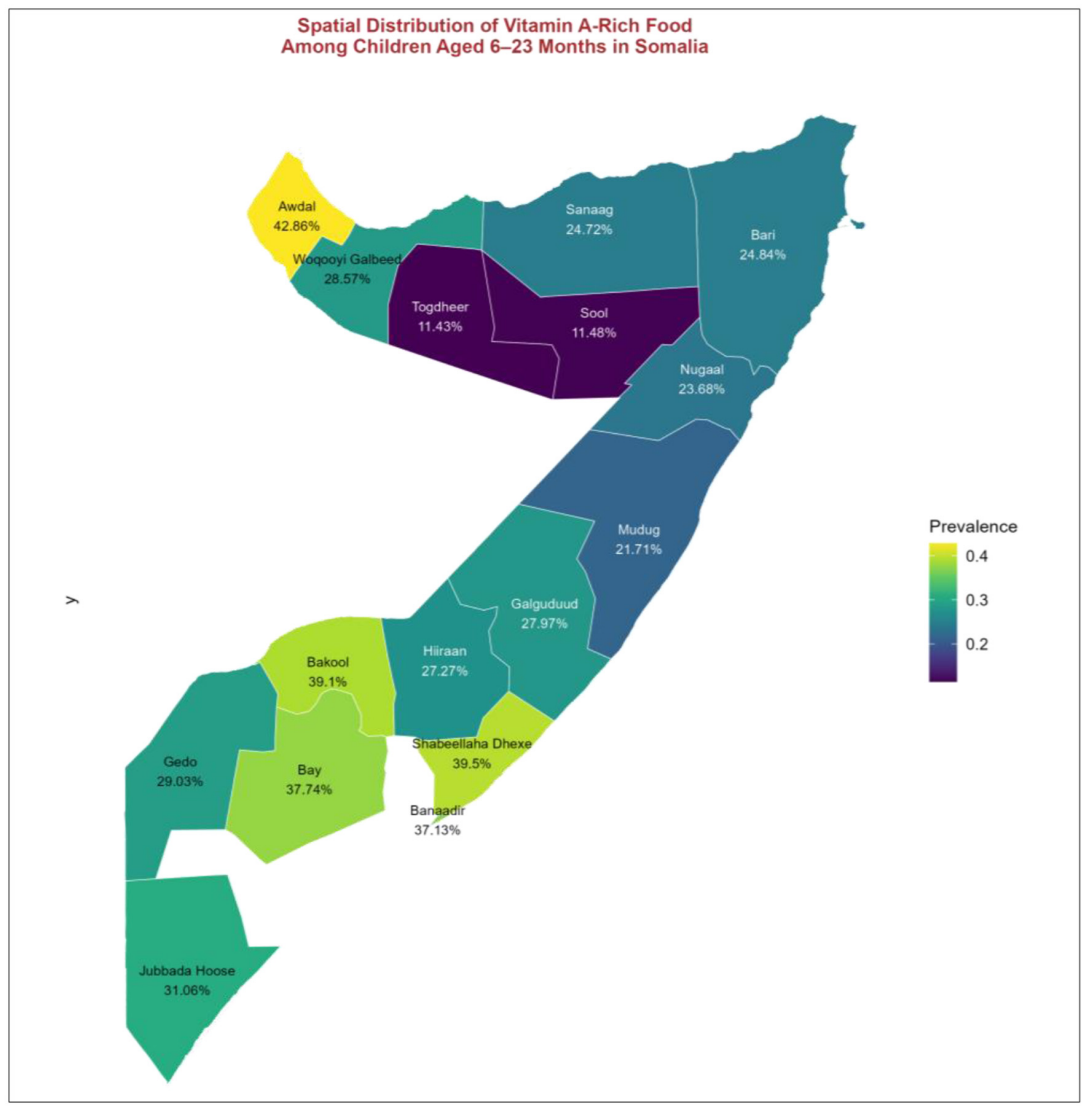
Spatial distribution of vitamin A-rich food consumption in Somalia.

In addition, Figure 1 illustrated the regional variations in vitamin A-rich food consumption among children aged 6–23 months in Somalia. The highest consumption was observed in Awdal (42.86%), Shabeellaha Dhexe (39.5%), and Bakool (39.1%), indicating better access to vitamin A-rich foods in these areas. In contrast, the lowest consumption was recorded in Togdheer (11.43%) and Sool (11.48%), suggesting potential disparities in dietary intake across regions. Geographically, southern regions such as Bay, Bakool, and Gedo exhibited relatively higher consumption levels, while northern regions, including Togdheer, Sool, Sanaag, and Nugaal, showed lower rates. The color gradient on the map visually highlighted these disparities, with yellow-green regions representing higher consumption levels and purple-blue regions indicating lower intake.

## Consumption patterns of vitamin A-rich foods among young children

**Figure 2:**
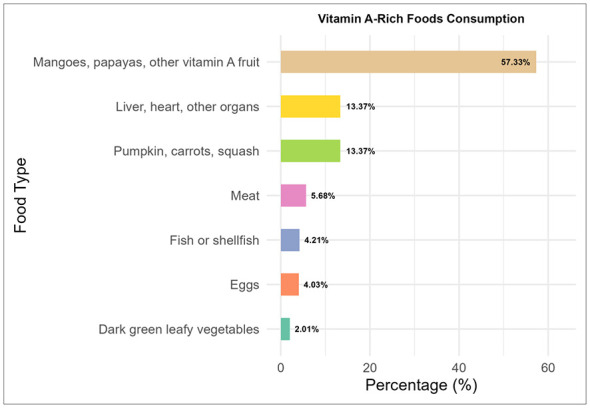
Vitamin A-rich food consumption patterns.

As shown in Figure 2, among the 546 children aged 6–23 months who were reported to have consumed at least one of the seven specified vitamin A-rich foods in the past 24 h, the most commonly consumed were vitamin A-rich fruits such as mangoes and papayas, with 313 children (57.33%) having eaten them. Pumpkin, carrots, and squash, along with organ meats like liver and heart, were each consumed by 73 children (13.37%). Meat (beef, lamb, chicken, etc.) consumption was reported for 31 children (5.68%), while fish or shellfish were eaten by 23 children (4.21%). Eggs were consumed by 22 children (4.03%), and dark green leafy vegetables had the lowest intake, with only 11 children (2.01%) reported to have eaten them. These patterns highlighted notable variations in the dietary patterns of young children in Somalia, with a strong reliance on vitamin A-rich fruits compared to other sources of essential nutrients.

## Demographic and socioeconomic characteristics of respondents

As shown in Table 3, in this study, Banadir region was the most represented (12.89%), while Togdheer had the smallest share (1.90%). Notably, regions such as Bari (8.75%), Lower Juba (8.75%), and Mudug (8.27%) also had significant representation. Regarding residence type, the majority of respondents lived in urban areas (44.26%), followed by nomadic populations (28.82%) and rural areas (26.92%). Wealth distribution across five quintiles revealed that 28.66% of the population fell within the lowest wealth quintile, followed by the second quintile (23.27%), middle (20.83%), fourth (16.26%), and the highest (10.98%). This distribution indicated that a significant portion of the population (over 50%) belonged to the lower wealth categories, highlighting economic disparities. In terms of household head characteristics, 67.32% of household heads were female, while 32.68% were male. Regarding age, 38.34% of household heads were aged between 21 and 40 years, while 38.23% were aged 41 years and above, and 23.44% were 20 years or younger. Education levels among husbands showed that 76.89% never attended school, while 23.11% had some level of education. Similarly, among mothers, 83.47% had no formal education, while 13.05% attained primary education, and only 3.48% reached secondary or higher education. Regarding access to media, the majority of mothers (89.56%) did not have access, while only 10.44% had media access. Concerning fertility, 43.72% of mothers had given birth to 1–3 children, 36.27% to 4–6 children, and 20.01% to 7 or more children. Regarding maternal health services, 67.81% of mothers reported not attending any antenatal care (ANC) visits during pregnancy. Only 6.96% had one ANC visit, 10.01% had two visits, and 15.23% had three or more visits. Place of delivery findings indicated that 82.16% of births occurred at home, while only 17.84% took place in health facilities. Regarding child demographics, 53.13% of children were male, while 46.87% were female. The birth order of children revealed that 12.45% were first-born, 46.00% were 2nd to 4th born, and 41.54% were 5th-born or higher. In terms of child age, the largest group was 12–17 months (49.05%), followed by 6–11 months (34.48%), and the smallest group was 18–23 months (16.48%). Finally, breastfeeding status showed that 64.93% of mothers were currently breastfeeding, while 35.07% were not breastfeeding at the time of the interview.

**Table 3:** Demographic and socioeconomic characteristics of respondents.

Variable	Levels	Frequency	Percentage
Region	Awdal	49	2.66
	Marodijeh	84	4.57
	Togdheer	35	1.90
	Sool	61	3.32
	Sanaag	89	4.84
	Bari	161	8.75
	Nugaal	152	8.27
	Mudug	152	8.27
	Galgaduud	118	6.42
	Hiraan	88	4.79
	Middle Shabelle	119	6.47
	Banadir	237	12.89
	Bay	53	2.88
	Bakool	156	8.48
	Gedo	124	6.74
	Lower Juba	161	8.75
Type of residence	Rural	495	26.92
	Urban	814	44.26
	Nomadic	530	28.82
Wealth quantile	Lowest	527	28.66
	Second	428	23.27
	Middle	383	20.83
	Fourth	299	16.26
	Highest	202	10.98
Sex of household head	Female	1,238	67.32
	Male	601	32.68
Age of household head	20 or less	431	23.44
	21- 40	705	38.34
	41 and above	703	38.23
Husband ever attended school	No	1,414	76.89
	Yes	425	23.11
Mothers age group	15–19	141	7.67
	20–24	486	26.43
	25–29	549	29.85
	30–34	344	18.71
	35–39	247	13.43
	40–49	72	3.92
Mother's education	No education	1,535	83.47
	Primary	240	13.05
	Secondary or higher	64	3.48
Media access	No	1,647	89.56
	Yes	192	10.44
Total children ever born	1–3	804	43.72
	4–6	667	36.27
	7 or more	368	20.01
Number of ANC visits during pregnancy	0	1,247	67.81
	1	128	6.96
	2	184	10.01
	3 or more	280	15.23
Place of delivery	Home	1,511	82.16
	Health center	328	17.84
Sex of child	Male	977	53.13
	Female	862	46.87
Birth order	First	229	12.45
	2nd to 4th	846	46.00
	≥ 5th	764	41.54
Age in months	6–11	634	34.48
	12–17	902	49.05
	18–23	303	16.48
Currently breastfeeding	Yes	1,194	64.93
	No	645	35.07

## Bivariate analysis: chi-square test results

As shown in Table 4, the chi-square (χ^2^) test results revealed significant associations between various background characteristics and the consumption of vitamin A-rich foods among children aged 6–23 months. Regional disparities in vitamin A intake were evident. The lowest consumption rates were observed in Sool (11.48%) and Togdheer (11.43%), while Middle Shabelle (39.50%) and Banadir (37.13%) had the highest. These differences may have stemmed from regional variations in food availability, nutritional awareness, or access to health and nutrition programs. Household size also played a role, with children from larger households (seven or more children) having a higher consumption rate (33.42%) than those from smaller households (1–3 children: 26.87%). This suggested that larger families may possess greater nutritional knowledge or prioritize vitamin A-rich foods. Economic status showed a strong correlation with vitamin A intake. Children from the lowest wealth quintile had the lowest consumption rate (14.80%), while those in the highest wealth group had the highest intake (53.47%). This likely reflected disparity in food affordability and access to diverse, nutrient-rich diets. Antenatal care (ANC) visits emerged as a significant factor. Children whose mothers had no ANC visits had a consumption rate of 24.22%, while those whose mothers attended three or more visits had nearly double the intake (46.79%). This highlighted the potential role of ANC in improving maternal awareness and access to nutritional resources. Maternal education was another crucial determinant. Children of mothers with secondary or higher education had significantly higher vitamin A intake (54.69%) compared to those whose mothers had no formal education (26.32%). Similarly, paternal education influenced dietary intake, with children of fathers who had attended school showing higher consumption (48.92%) compared to those whose fathers had no formal education (24.19%). Media access also played a decisive role, as children from households with access to media had significantly higher consumption (55.73%) than those without (0.53%). This suggested that exposure to health and nutrition messages through media may positively impact dietary choices. The age of the household head was another factor, with children in households headed by individuals aged 41–50 years having the highest vitamin A intake (46.34%), whereas those in households with a head younger than 21 years had the lowest (27.15%). Child's age was significantly associated with vitamin A intake. Children aged 12–17 months had the highest consumption (38.58%), while those aged 6–11 months had the lowest (17.82%). This may indicate that older children were more likely to be introduced to solid foods rich in vitamin A. Place of delivery influenced dietary intake, as children born in healthcare facilities had a higher consumption rate (43.90%) compared to those born at home (26.60%), underscoring the role of healthcare settings in promoting better nutrition. Breastfeeding status also mattered; non-breastfed children had a higher vitamin A intake (35.81%) compared to those currently breastfeeding (26.38%). This may suggest that non-breastfed children were introduced to a more diverse diet earlier. Birth order further influenced vitamin A consumption. First-born children had the lowest intake (20.52%), while those with a birth order of fifth or higher had the highest (33.25%). This pattern may reflect increased parental experience and improved feeding practices over time.

**Table 4:** Bivariate analysis.

Variable	Vitamin A rich food consumption among children aged 6–23 months		Chi-square (*X*^2^)	*P*-value
	No	Yes		
Region				
Awdal	28 (57.14)	21(42.86)	50.15	0.0001
Marodijeh	60 (71.43)	24 (28.57)		
Togdheer	31 (88.57)	4 (11.43)		
Sool	54 (88.52)	7 (11.48)		
Sanaag	67 (75.28)	22 (24.72)		
Bari	121 (75.16)	40 (24.84)		
Nugaal	116 (76.32)	36 (23.68)		
Mudug	119 (78.29)	33 (21.71)		
Galgaduud	85 (72.03)	33 (27.97)		
Hiraan	64 (72.73)	24 (27.27)		
Middle Shabelle	72 (60.50)	47 (39.50)		
Banadir	149 (62.87)	88 (37.13)		
Bay	33 (62.26)	20 (37.74)		
Bakool	95 (60.90)	61 (39.10)		
Gedo	88 (70.97)	36 (29.03)		
Lower Juba	111 (68.94)	50 (31.06)		
Place of residence				
Rural	353 (71.31)	142 (28.69)	0.33	0.846
Urban	570 (70.02)	244 (29.98)		
Nomadic	370 (69.81)	546 (30.19)		
Wealth quantiles				
Lowest	449 (85.20)	78 (14.80)	160.00	0.0001
Second	339 (79.21)	89 (20.79)		
Middle	233 (60.84)	150 (39.16)		
Fourth	178 (59.53)	121 (40.47)		
Highest	94 (46.53)	108 (53.47)		
Sex of household head				
Male	863 (69.71)	375 (20.29)	0.6549	0.418
Female	430 (71.55)	171 (28.45)		
Age of household head				
20 or less	314 (72.85)	117 (27.15)	3.45	0.178
21- 40	479 (67.94)	226 (32.06)		
41 and above	500 (71.31)	203 (28.88)		
Husband ever attended school				
No	1,072 (75.81)	342 (24.19)	88.77	0.0001
Yes	221 (52.00)	204 (48.00)		
Mothers age				
15–19	109 (77.30)	32 (22.70)	8.86	0.115
20–24	251 (72.22)	135 (27.88)		
25–29	376 (68.49)	173 (31.51)		
30–34	231 (67.15)	113 (32.85)		
	No	Yes		
35–39	170 (68.83)	77 (31.77)		
40–49	56 (77.78)	16 (22.22)		
Mother's education				
No education	1,131 (73.68)	404 (26.32)	53.01	0.0001
Primary	133 (55.42)	107 (44.58)		
15.6-7.2,-13.6498ptSecondary or higher	29 (45.31)	35 (54.69)		
Media Access				
No	1,208 (73.35)	43 (0.53)	69.63	0.0001
Yes	85 (44.27)	107 (55.73)		
Total children ever born				
1–3	588 (73.13)	216 (26.87)	6.11	0.0472
4–6	460 (68.97)	207 (31.03)		
7 or more	245 (66.58)	123 (33.42)		
Number of antenatal visits during pregnancy				
Zero	945 (75.78)	302 (24.22)	63.63	0.0001
One	80 (62.50)	48 (37.50)		
Two	119 (64.67)	65 (35.33)		
Three or more	149 (53.21)	131 (46.79)		
Place of delivery				
Home	1,109 (73.40)	402 (26.60)	38.60	0.0001
Health center	184 (56.10)	144 (43.90)		
Sex of child				
Male	697 (71.34)	280 (28.66)	1.06	0.303
Female	596 (69.14)	266 (30.86)		
Birth order				
First	182 (79.48)	47 (20.52)	14.06	0.0009
2nd−4th	601 (71.04)	245 (28.96)		
≥5th	510 (66.75)	254 (33.25)		
Age in months				
6–11	521 (82.18)	113 (17.82)	77.31	0.0001
12–17	554 (61.42)	348 (38.58)		
18–23	218 (71.95)	85 (28.05)		
Currently breastfeeding				
Yes	879 (73.62)	315 (26.38)	17.85	0.0001
No	414 (64.19)	231 (35.81)		

## Inequalities

In this study, the socioeconomic inequalities in vitamin A-rich food consumption among children aged 6–23 months in Somalia were assessed using the Concentration Index (CIX) approach. Both the Relative CIX and the Modified CIX were computed to capture the extent of inequality while accounting for potential biases associated with outcome prevalence. As shown in Table 5, the findings indicated that wealth-related inequality in vitamin A-rich food consumption was substantial. The Relative CIX for wealth was 0.2533 (*SE* = 0.0195, ^*^p^*^ < 0.001), suggesting that consumption was disproportionately concentrated among wealthier households. The Modified CIX for wealth, which adjusted for the high prevalence of the outcome, was 0.2472, reinforcing the observed inequality. Similarly, education-related inequality was assessed, with the Relative CIX computed at 0.1438 (*SE* = 0.0220, ^*^p^*^ < 0.001), indicating that children from more educated households were more likely to consume vitamin A-rich foods. The Modified CIX for education was 0.0963 (*SE* = 0.0132, ^*^p^*^ < 0.001), reflecting a lower magnitude of inequality after adjusting for the distribution of the outcome variable.

**Table 5:** Concentration index.

Variable	Type	Coefficient	Standard error	*P*-value	95% CI
Wealth	Relative CIX	0.2533	0.0195	0.001	0.2151–0.2916
	Modified CIX	0.2472	0.0194	0.001	—
Education	Relative CIX	0.1438	0.0220	0.001	0.1007–0.1868
	Modified CIX	0.0963	0.0132	0.001	—

CIX, concentration index; CI, confidence interval.

**Figure 3:**
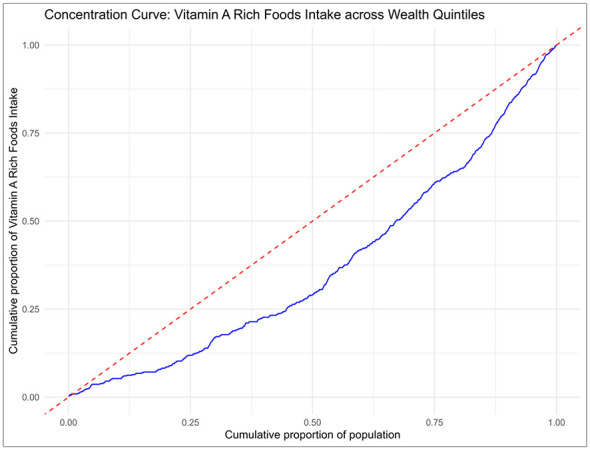
Concentration curve across wealth quintiles.

**Figure 4:**
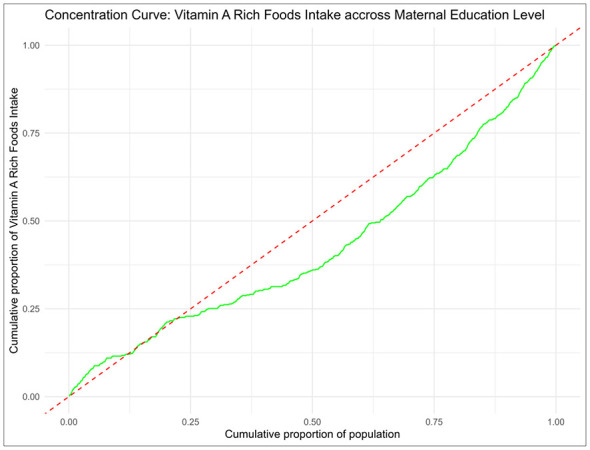
Concentration curve across maternal education level.

As shown in Figure 3, findings from the concentration curve revealed a clear socioeconomic disparity in vitamin A-rich food consumption among Somali children aged 6–23 months based on wealth index. The curve consistently fell below the equality line, indicating that access to these essential nutrients was disproportionately concentrated among children from wealthier households, aligning with the concentration index. Similarly, Figure 4 presents the concentration curve based on maternal education level, showing that children of more educated mothers had greater access to vitamin A-rich foods. The curve again fell below the equality line, reinforcing the pattern of inequality in nutrient access associated with maternal education.

## Model comparison

As shown in Table 6 among the four models tested, Model III demonstrated the best fit, as indicated by the lowest AIC (1953.13) and the highest log-likelihood (-937.56). A lower AIC signified a more optimal balance between model complexity and goodness of fit, while a higher log-likelihood indicated that the model better explained the observed data. The intraclass correlation coefficient (ICC) decreased substantially from 0.22 in the empty model to 0.02 in Model III, suggesting that cluster-level variations accounted for only 2% of the variability in vitamin A-rich food consumption. Additionally, the random intercept's variance declined from 0.94 in the empty model to 0.06 in Model III, highlighting a significant reduction in unexplained variation at the cluster level. Given these findings, Model III was selected as the final model, as it offered the best trade-off between model fit, variance reduction, and theoretical interpretability in explaining vitamin A-rich food consumption.

**Table 6:** Model comparison metrics.

Criteria	Model 0 (empty model)	Model I	Model II	Model III
AIC	2,181.07	1,968.96	2,055.03	1,953.13
BIC	2,192.10	2,084.81	2,165.37	2,168.29
ICC	0.22	0.01	0.06	0.02
Log-likelihood	−1,088.54	−963.48	−1,007.52	−937.56
Variance	0.94	0.05	0.19	0.06

AIC, information criterion; BIC, Bayesian information criterion; ICC, intra-cluster correlation.

## Multilevel binary logistic regression

As shown in Table 7, the results from Model III indicated significant regional disparities in the consumption of vitamin A-rich foods among children aged 6–23 months, with children in Sool (*AOR* = 0.36, CI: 0.12–1.04), Sanaag (*AOR* = 0.30, CI: 0.12–0.71), and Mudug (*AOR* = 0.26, CI: 0.12–0.57) being significantly less likely to consume vitamin A-rich foods compared to those in Awdal. Household wealth status emerged as a strong predictor, as children from middle-income (*AOR* = 2.75, CI: 1.89–4.01) and high-income households (*AOR* = 3.60, CI: 2.23–5.78) had substantially higher odds of consuming these foods compared to those from low-income households. Media access also played a significant role, with children from households with regular media exposure having more than twice the odds of consuming vitamin A-rich foods (*AOR* = 2.05, CI: 1.41–2.98). Additionally, paternal education was positively associated with dietary diversity, as children whose fathers had formal education were more likely to consume vitamin A-rich foods (*AOR* = 1.83, CI: 1.35–2.49). Maternal healthcare utilization was another important determinant, with children of mothers who attended at least one ANC visit being more likely to consume vitamin A-rich foods (*AOR* = 1.69, CI: 1.09–2.62), suggesting that healthcare access may improve maternal knowledge and practices regarding child nutrition. Child age was also a significant factor, with older children (12–17 months) having nearly three times the odds of consuming vitamin A-rich foods compared to younger children (*AOR* = 2.94, CI: 2.22–3.89). Birth order influenced dietary intake as well, with children of higher birth order (≥5) having greater odds of consuming vitamin A-rich foods (*AOR* = 2.12, CI: 1.21–3.72). In addition, community education was also a significant determinant of children's consumption of vitamin A-rich foods. Children from communities with lower overall education levels had lower odds of consuming these foods (*AOR* = 0.63, CI: 0.42–0.93). However, factors such as place of residence (urban vs. rural), breastfeeding status, and place of delivery did not show significant associations, indicating that economic, educational, and healthcare-related factors played a more dominant role in shaping dietary practices.

**Table 7:** Multilevel binary logistic regression.

Variable	Model I OR (95% CI)	Model II *AOR* (95% CI)	Model III *AOR* (95% CI)
Region			
Awdal		1 (Ref)	1 (Ref)
Marodijeh		0.64 (0.29–1.44)	0.79 (0.34–1.82)
Togdheer		0.40 (0.11–1.44)	0.44 (0.12–1.62)
Sool		0.38 (0.13–1.09)	0.41 (0.14–1.18)
Sanaag		0.36 (0.16–0.82)^*^	0.31 (0.13–0.73)^*^
Bari		0.31 (0.15–0.65)^*^	0.33 (0.15–0.71)^*^
Nugaal		0.32 (0.15–0.68)^*^	0.33 (0.15–0.71)^*^
Mudug		0.28 (0.13–0.59)^*^	0.25 (0.11–0.55)^*^
Galgaduud		0.38 (0.18–0.82)^*^	0.44 (0.20–0.97)^*^
Hiraan		0.49 (0.22–1.10)	0.46 (0.20v1.06)
Middle Shabelle		0.67 (0.321.41)	0.70 (0.32v1.53)
Banadir		0.72 (0.36–1.41)	0.67 (0.33–1.37)
Bay		0.38 (0.15–0.94)^*^	0.43 (0.17–1.10)
Bakool		0.58 (0.28–1.20)	0.58 (0.27–1.23)
Gedo		0.53 (0.25–1.14)^*^	0.55 (0.25–1.21)
Lower Juba		0.28 (0.13–0.58)^*^	0.32 (0.15–0.68)^*^
Community media exposure			
High media access		1 (Ref)	1 (Ref)
Low media access		1.48 (0.98–2.23)	1.13 (0.77–1.65)
Community education			
High literacy		1 (Ref)	1 (Ref)
Low literacy		0.51 (0.33–0.77)^*^	0.63 (0.42–0.93)^*^
Community poverty			
High poverty		1 (Ref)	1 (Ref)
Low poverty		0.37 (0.23–0.60)^*^	0.71 (0.40–1.23)
Wealth quantiles			
Lowest	1 (Ref)		1 (Ref)
Second	1.31 (0.92–1.85)		1.16 (0.80–1.69)
Middle	2.78 (1.96–3.94)^*^		1.79 (1.14–2.81)^*^
Fourth	2.43 (1.65–3.57)^*^		1.53 (1.30–3.85)
Highest	3.39 (2.16–5.32)^*^		2.23 (1.30–3.85)^*^
Husband ever attended school			
No	1 (Ref)		1 (Ref)
Yes	1.70 (1.28–2.26)^*^		1.69 (1.26–2.26)^*^
Mother's education			
No education	1 (Ref)		1 (Ref)
Primary	1.04 (0.74–1.47)		1.04 (0.73–1.48)
Secondary or higher	1.12 (0.60–2.07)		1.11 (0.59–2.09)
Media access			
No	1 (Ref)		1 (Ref)
Yes	2.00 (1.39–2.87)^*^		1.88 (1.28–2.77)^*^
Total children ever born			
1–3	1 (Ref)		1 (Ref)
4–6	1.05 (0.74–1.49)		1.00 (0.70–1.44)
7 or more	1.02 (0.63–1.65)		0.99 (0.60–1.62)
Number of antenatal visits during pregnancy			
Zero	1 (Ref)		1 (Ref)
One	1.68 (1.10–2.57)^*^		1.62 (1.04–2.51)^*^
Two	1.11 (0.76–1.61)		1.06 (0.72–1.55)
Three or more	1.74 (1.26–2.40)^*^		1.55 (1.11–2.17)^*^
Place of delivery			
Home	1 (Ref)		1 (Ref)
Health center	1.07 (0.78–1.46)		0.98 (0.71–1.36)
Birth order			
First	1 (Ref)		1 (Ref)
2nd to 4th	1.90 (1.26–2.87)^*^		1.85 (1.22–2.81)^*^
≥5th	2.08 (1.20–3.61)^*^		1.95 (1.11–3.43)^*^
Age in months			
6–11	1 (Ref)		1 (Ref)
12–17	2.92 (2.22–3.84)^*^		2.89 (2.18–3.82)^*^
18–23	2.38 (1.65–3.43)^*^		2.60 (1.78–3.80)^*^
Currently breastfeeding			
Yes	1 (Ref)		1 (Ref)
No	1.23 (0.97–1.56)		1.22 (0.96–1.56)

AOR, adjusted odds ratio; CI, confidence interval; Ref, reference category.

^*^p-value < 0.05.

### Cluster-level findings

Community education (low literacy) was significantly associated with lower odds of vitamin A-rich food consumption (*AOR* = 0.63, 95% CI: 0.42–0.93), while community media exposure and community poverty were not significant in the fully adjusted model.

## Discussion

This study examined the determinants and socioeconomic inequalities in vitamin A-rich food consumption among children aged 6–23 months in Somalia, utilizing data from the 2020 Somalia Health and Demographic Survey (SHDS). The findings revealed a complex interplay of factors influencing dietary intake, with significant regional disparities and socioeconomic inequalities. A key finding was the strong association between socioeconomic status and vitamin A consumption, aligning with evidence from other low- and middle-income countries (LMICs) (14, 15, 23). Children from wealthier households demonstrated significantly higher consumption rates compared to those from the poorest quintile, consistent with findings from Ethiopia where similar wealth-related inequalities were observed (36), suggesting that affordability and access to diverse, nutrient-rich diets remain significant barriers for low-income families in Somalia (22). The study's Concentration Index further reinforced this point, with the relative CIX for wealth (0.2533, ^*^p^*^ < 0.001) confirming substantial wealth-related inequality. Similarly, maternal education emerged as a crucial determinant (Relative CIX = 0.1438, ^*^p^*^ < 0.001), consistent with studies from other developing nations (15, 23, 24, 26, 27), as children whose mothers had secondary or higher education exhibited significantly higher vitamin A intake compared to those whose mothers had no formal education. Children whose mothers attended at least one ANC visit had significantly higher odds of consuming vitamin A-rich foods, underscoring the critical role of maternal health services in shaping child feeding practices; this dose-response relationship persisted even after adjusting for maternal education and household wealth, suggesting ANC exerts an independent effect through health information and strengthened maternal self-efficacy, aligning with evidence from Ethiopia, Bangladesh, India, and Indonesia (15, 26, 27, 35). However, with 67.81% of women receiving no ANC during pregnancy, this represents a critical missed opportunity for nutrition promotion. Marked regional disparities were evident, with children in Sanaag, Bari, Nugaal, Mudug, Galgaduud, and Lower Juba having substantially lower consumption rates compared to Awdal, likely reflecting a complex interplay of geographical, cultural, and infrastructural factors—including predominantly nomadic pastoralist livelihoods in northern regions and the compounded effects of prolonged conflict and displacement in southern Somalia (39). Community-level education also emerged as a significant determinant: children living in communities with low literacy had 37% lower odds of consuming vitamin A-rich foods (*AOR* = 0.63, 95% CI: 0.42–0.93) even after adjusting for individual-level maternal education, suggesting that low community literacy may reflect broader contextual factors including collective health literacy and social norms around child feeding. The finding that older children (12–17 months) had nearly three times higher odds of consuming vitamin A-rich foods compared to infants aged 6–11 months was consistent with the natural progression of complementary feeding, aligning with studies from Ethiopia (35) and Bangladesh (15). The positive association between higher birth order (≥5th child) and vitamin A-rich food consumption may reflect that multiparous mothers develop improved child feeding practices through accumulated experience (36). Contrary to studies where urban or rural residence was a significant predictor of dietary intake (21), this study found no statistically significant association, which may be attributed to the unique context of Somalia where both rural and nomadic populations face similarly severe constraints in accessing diverse and nutritious foods. Given the exceptionally low levels of vitamin A-rich food consumption observed (29.7% weighted prevalence), addressing vitamin A deficiency through dietary diversification alone may not be currently feasible in Somalia; thus, integrated strategies combining long-term efforts to improve infant and young child feeding (IYCF) practices with continued, high-coverage implementation of vitamin A supplementation programs are imperative to protect vulnerable populations (30, 31).

### Limitations and future directions

The current study relied on cross-sectional data, which limits the ability to establish causal relationships between the identified determinants and vitamin A consumption. Future longitudinal studies are needed to explore the temporal relationships between these factors and dietary outcomes. Furthermore, the study's reliance on self-reported dietary data may be subject to recall bias. Future research could benefit from using more objective measures of dietary intake, such as food frequency questionnaires or 24-h dietary recalls.

Postnatal care (PNC) was not included in the analysis because the SHDS 2020 did not collect data on PNC visits for the full sample of children aged 6–23 months. PNC data were only available for a subset of recent births (< 2 months), which would have substantially reduced the analytic sample and introduced selection bias. We acknowledge this as a limitation and recommend that future surveys include PNC indicators for all children under 2 years. Future research should also focus on longitudinal studies to assess the long-term impact of these interventions.

### Practical implications for policy and programs

Our findings offer several actionable insights for policymakers and program implementers in Somalia:

#### Targeted vs. universal interventions

The substantial wealth-related inequality suggests that universal nutrition programs will not close the gap. We recommend that the Ministry of Health prioritize the lowest two wealth quintiles for vitamin A supplementation outreach and complementary feeding support, potentially using existing community health worker networks.

#### Leveraging ANC contacts

With 67.8% of women receiving no ANC, strengthening ANC coverage is critical. However, for women who do attend (15.2% attended ≥3 visits), each visit represents an opportunity for nutrition counseling. We recommend integrating a standardized vitamin A and IYCF counseling module into all ANC visits, delivered by trained midwives or nurses.

#### Region-specific strategies

The very low consumption in Togdheer (11.4%), Sool (11.5%), and Mudug (21.7%) calls for intensified supplementation campaigns, possibly through mobile clinics. In contrast, regions with moderate consumption (e.g., Awdal, 42.9%) may benefit more from agricultural diversification and home gardening programs to sustain dietary sources.

#### Media and education

The strong effect of media access (*AOR* = 2.05) suggests that radio-based nutrition campaigns—which are low-cost and can reach nomadic populations—should be expanded. Similarly, community-level education interventions (e.g., mother-to-mother support groups in low-literacy areas) could help address the contextual effect of low community education.

### Revised recommendations section

Based on these findings, we recommend:

#### For the ministry of health

Immediately prioritize vitamin A supplementation campaigns in Togdheer, Sool, and Mudug, targeting 6–59 month old children with at least 90% coverage. Integrate IYCF counseling into all ANC contacts using a standardized checklist.

#### For donors and NGOs

Fund mobile nutrition services for nomadic populations in Sanaag, Bari, and Nugaal. Support radio-based nutrition behavior change communication campaigns in local languages.

#### For future research

Conduct implementation research to test the effectiveness of cash transfers conditional on vitamin A-rich food purchases among lowest-quintile households. Evaluate the impact of mother-to-mother support groups on child dietary diversity in low-literacy communities.

## Conclusion

This study provides valuable insights into the determinants and socioeconomic inequalities in vitamin A-rich food consumption among children in Somalia. The findings highlight the importance of addressing socioeconomic barriers, promoting maternal education and healthcare access, and implementing targeted interventions to improve child nutrition. While improving dietary diversity through food-based approaches remains the ultimate goal, the low levels of intake observed in this study underscore the continued importance of vitamin A supplementation as an immediate strategy to prevent deficiency and reduce child mortality in Somalia. Integrated strategies that combine efforts to improve infant and young child feeding practices with sustained, high-coverage supplementation programs are essential for achieving meaningful and lasting improvements in child nutrition. By addressing these factors, policymakers and practitioners can contribute to reducing vitamin A deficiency and improving the health and wellbeing of Somali children.

## Data Availability

Publicly available datasets were analyzed in this study. This data can be found here: https://microdata.nbs.gov.so/index.php/catalog/50.

## References

[1] Lucha TA Engida TA Mengistu AK. Assessing the potential determinants of national vitamin A supplementation among children aged 6–35 months in Ethiopia: further analysis of the 2019 Ethiopian mini demographic and health survey. BMC Pediatr. (2022) 22:439. doi: 10.1186/s12887-022-03499-5 35864488 PMC9306167

[2] Marriott BP White A Hadden L Davies JC Wallingford JC. World health organization (WHO) infant and young child feeding indicators: associations with growth measures in 14 low-income countries. Matern Child Nutr. (2012) 8:354–70. doi: 10.1111/j.1740-8709.2011.00380.x 22171937 PMC6860880

[3] FAO World Health Organization. Vitamin and mineral requirements in human nutrition Second edition. Geneva: World Health Organization [Internet]. (1998) 1–20. Available online at: www.who.org

[4] Ramakrishnan U. Prevalence of micronutrient malnutrition worldwide. Nutr Rev. (2002) 60:S46–52. doi: 10.1301/00296640260130731 12035858

[5] Zeitlin MF Megawangi R Kramer EM Armstrong HC. Mothers' and children's intakes of vitamin A in rural Bangladesh. Am J Clin Nutr. (1992) 56:136–47. doi: 10.1093/ajcn/56.1.136 1609750

[6] Ramakrishnan U Martorell R Latham MC Abel R. Dietary vitamin A intakes of preschool-age children in South India. J Nutr. (1999) 129:2021–7. doi: 10.1093/jn/129.11.2021 10539779

[7] Sommer A. Vitamin A deficiency and its consequences: a field guide to detection and control. Geneva: World Health Organization. (1995) 100.

[8] Englberger L Marks GC Fitzgerald MH Timothy J. Vitamin A intake and factors influencing it amongst children and caretakers in Kosrae, Micronesia. Ecol Food Nutr. (2005) 44:307–38. doi: 10.1080/03670240500187369

[9] Stevens GA Bennett JE Hennocq Q Lu Y De-Regil LM Rogers L . Trends and mortality effects of vitamin A deficiency in children in 138 low-income and middle-income countries between 1991 and 2013: a pooled analysis of population-based surveys. Lancet Glob Heal. (2015) 3:e528–36. doi: 10.1016/S2214-109X(15)00039-X 26275329

[10] World Health Organization. Global prevalence of vitamin A deficiency in populations at risk 1995-2005 : WHO global database on vitamin A deficiency. WHO Iris [Internet]. (2009) p. 55. Available online at: http://apps.who.int//iris/handle/10665/44110 (Accessed April 15, 2026).

[11] Harika R Faber M Samuel F Mulugeta A Kimiywe J Eilander A. Are low intakes and deficiencies in iron, vitamin A, zinc, and iodine of public health concern in Ethiopian, Kenyan, Nigerian, and South African children and adolescents? Food Nutr Bull. (2017) 38:405–27. doi: 10.1177/0379572117715818 28682645

[12] Baye K Laillou A Seyoum Y Zvandaziva C Chimanya K Nyawo M. Estimates of child mortality reductions attributed to vitamin A supplementation in sub-Saharan Africa: scale up, scale back, or refocus? Am J Clin Nutr. (2022) 116:426–34. doi: 10.1093/ajcn/nqac082 35380631

[13] Vosti SA Kagin J Engle-Stone R Luo H Tarini A Clermont A . Strategies to achieve adequate vitamin A intake for young children: options for Cameroon. Ann N Y Acad Sci. (2020) 1465:161–80. doi: 10.1111/nyas.14275 31797386 PMC7187426

[14] Tiruneh SA Fentie DT Yigizaw ST Abebe AA Gelaye KA. Spatial distribution and geographical heterogeneity factors associated with poor consumption of foods rich in vitamin A among children age 6–23 months in Ethiopia: geographical weighted regression analysis. PLoS ONE. (2021) 16:e0252639. doi: 10.1371/journal.pone.0252639 34081718 PMC8174682

[15] Kundu S Das P Rahman MA Al Banna MH Fatema K Islam MA . Socio-economic inequalities in minimum dietary diversity among Bangladeshi children aged 6–23 months: a decomposition analysis. Sci Rep. (2022) 12:21712. doi: 10.1038/s41598-022-26305-9 36522494 PMC9755277

[16] Abrha T Girma Y Haile K Hailu M Hailemariam M. Prevalence and associated factors of clinical manifestations of vitamin a deficiency among preschool children in asgede-tsimbla rural district, north Ethiopia, a community based cross sectional study. Arch Public Heal. (2016) 74:1–8. doi: 10.1186/s13690-016-0122-3 PMC4790060 26977293

[17] Ezzati M Vander Hoorn S Lopez AD Danaei G Rodgers A Mathers CD . Comparative quantification of mortality and burden of disease attributable to selected risk factors. in Global Burden Disease Risk Factors. Washington (DC): The International Bank for Reconstruction and Development/The World Bank; New York: Oxford University Press. (2006) 573–601. 21250375

[18] Oyunga MA Grant FKE Omondi DO Ouedraogo H Levin C Low JW. Prevalence and predictors of vitamin A deficiency among infants in western Kenya using a cross-sectional analysis. African J Food, Agric Nutr Dev. (2016) 16:10765–86. doi: 10.18697/ajfand.73.16190

[19] Alemayehu T Haider J Habte D. Determinants of adolescent fertility in Ethiopia. Ethiop J Heal Dev. (2010) 2430–8. doi: 10.4314/ejhd.v24i1.62942

[20] Wolde M Tessema ZT. Determinants of good vitamin A consumption in the 12 East Africa countries using recent demographic and health survey. PLoS ONE. (2023) 18:e0281681. doi: 10.1371/journal.pone.0281681 36795660 PMC9934452

[21] Baek Y Chitekwe S. Sociodemographic factors associated with inadequate food group consumption and dietary diversity among infants and young children in Nepal. PLoS ONE. (2019) 14:e0213610. doi: 10.1371/journal.pone.0213610 30856209 PMC6411102

[22] Peeters A Blake MRC. Socioeconomic inequalities in diet quality: from identifying the problem to implementing solutions. Curr Nutr Rep. (2016) 5:150–9. doi: 10.1007/s13668-016-0167-5

[23] Ricardo LIC Gatica-Domínguez G Neves PAR Vaz J. dos S, Barros AJD, Wehrmeister FC. Sociodemographic inequalities in vegetables, fruits, and animal source foods consumption in children aged 6–23 months from 91 LMIC. Front Nutr. (2023) 10:1046686. doi: 10.3389/fnut.2023.1046686 36866060 PMC9972219

[24] Srivastava S Kumar S. Does socio-economic inequality exist in micro-nutrients supplementation among children aged 6–59 months in India? Evidence from national family health survey 2005–06 and 2015–16. BMC Public Health. (2021) 21:1–12. doi: 10.1186/s12889-021-10601-6 33740942 PMC7980608

[25] Wagstaff A O'Donnell O Van Doorslaer E Lindelow M. Analyzing Health Equity Using Household Survey Data: A Guide to Techniques and Their Implementation. Washington, DC: World Bank Publications. (2007).

[26] Tripathy A Bhati DK Srivastava S Mishra PS. Change in socioeconomic inequality in minimum dietary diversity among children aged 6–23 months in India: evidence from national family health survey. Child Indic Res. (2023) 16:1049–71. doi: 10.1007/s12187-022-10004-y

[27] Paramashanti BA Dibley MJ Alam A Huda TM. Wealth-and education-related inequalities in minimum dietary diversity among Indonesian infants and young children: a decomposition analysis. Glob Health Action. (2022) 15:2040152. doi: 10.1080/16549716.2022.2040152 35389332 PMC9004518

[28] Akhtar S Ahmed A Randhawa MA Atukorala S Arlappa N Ismail T . Prevalence of vitamin A deficiency in South Asia: causes, outcomes, and possible remedies. J Health Popul Nutr. (2013) 31:413. doi: 10.3329/jhpn.v31i4.19975 24592582 PMC3905635

[29] West KP Jr. Epidemiology and prevention of vitamin A deficiency disorders. Retin Biol Biochem Dis. 2015;505–27. doi: 10.1002/9781118628003.ch23

[30] Kassa G Mesfin A Gebremedhin S. Uptake of routine vitamin A supplementation for children in Humbo district, southern Ethiopia: community-based cross-sectional study. BMC Public Health. (2020) 20:1–8. doi: 10.1186/s12889-020-09617-1 33008352 PMC7532605

[31] Kundu S Rai B Shukla A. Prevalence and determinants of Vitamin A deficiency among children in India: Findings from a national cross-sectional survey. Clin Epidemiol Glob Heal. (2021) 11:100768. doi: 10.1016/j.cegh.2021.100768

[32] Kennedy ET Ruth O. Household and preschooler vitamin A consumption in southwestern Kenya. J Nutr. (1993) 123:841–6. doi: 10.1093/jn/123.5.841 8487095

[33] Tarwotjo I West KP Mele L Nur S Nendrawati H Kraushaar D . Determinants of community-based coverage: periodic vitamin A supplementation. Am J Public Health. (1989) 79:847–9. doi: 10.2105/AJPH.79.7.847 2735470 PMC1349663

[34] DNS Government of Somalia and Demographic Survey 2020. SHD Surv 2020 Somalia. (2020).

[35] Demsash AW Chereka AA Kassie SY Donacho DO Ngusie HS Tegegne MD . Spatial distribution of vitamin A rich foods intake and associated factors among children aged 6–23 months in Ethiopia: spatial and multilevel analysis of 2019 Ethiopian mini demographic and health survey. BMC Nutr. (2022) 8:77. doi: 10.1186/s40795-022-00573-0 35953835 PMC9367059

[36] Merid MW Aragaw FM Godana TN Kibret AA Alem AZ Asratie MH . Wealth-related inequality in vitamin A rich food consumption among children of age 6–23 months in Ethiopia; Wagstaff decomposition of the 2019 mini-DHS data. PLoS One. (2024) 19:e0302368. doi: 10.1371/journal.pone.0302368 39378195 PMC11460695

[37] Belay DG Wassie MM Alemu MB Merid MW Norman R Tessema GA. Socio-economic and spatial inequalities in animal sources of iron-rich foods consumption among children 6–23 months old in Ethiopia: A decomposition analysis. PLOS Glob Public Heal. (2024) 4:e0003217. doi: 10.1371/journal.pgph.0003217 38753686 PMC11098381

[38] Erreygers G Van Ourti T. Measuring socioeconomic inequality in health, health care and health financing by means of rank-dependent indices: a recipe for good practice. J Health Econ. (2011) 30:685–94. doi: 10.1016/j.jhealeco.2011.04.004 21683462 PMC3158909

[39] Tiruneh FN Tenagashaw MW Asres DT Cherie HA. Associations of early marriage and early childbearing with anemia among adolescent girls in Ethiopia: a multilevel analysis of nationwide survey. Arch Public Heal [Internet]. (2021) 79:91. doi: 10.1186/s13690-021-00610-7 34082813 PMC8173845

